# Weight stigma among diverse ethnic groups with obesity in the U.S.: the USA-OBESTIGMA study

**DOI:** 10.1038/s41366-026-02028-z

**Published:** 2026-02-14

**Authors:** Rodolfo J. Galindo, Giuliana Arevalo, Zohyra Zabala, Ina Flores, Diana Soliman, Bobak Moazzami, Ammar Rashied, Albert Lecube, Guillermo E. Umpierrez

**Affiliations:** 1 Division of Endocrinology, University of Miami Miller School of Medicine, Miami, FL USA; 2 Division of Endocrinology, Emory University School of Medicine, Atlanta, GA USA; 3 Rollins School of Public Health, Emory University, Atlanta, GA USA; 4 Hospital Universitari Vall de’Hebron. Vall de’Hebron Research Institute (VHIR). CIBERdem, Barcelona, Spain; 5 Spanish Society of Obesity (SEEDO), Madrid, Spain

**Keywords:** Obesity, Epidemiology

## Abstract

**Background:**

The evidence on weight-related stigmatization among U.S. Hispanic (HISP), non-Hispanic White (NHW), and non-Hispanic Black (NHB) adults with obesity is limited. We compare levels of prejudices, stigma, and internalization of negative bias across diverse minority groups using validated surveys.

**Methods:**

Observational study conducted at two academic centers, including adults ( ≥ 18 years of age) with body mass index (BMI) ≥ 30 kg/m^2^. We assessed 1) weight-related aversion/prejudices using Anti-Fat Attitudes (AFA) survey, 2) stigmatizing experiences with the Brief Stigmatizing Situations Inventory (SSI-B), and 3) internalization of negative weight bias with the Weight Bias Internalization Scale (WBIS) survey.

**Results:**

Among 296 participants (age 54.9 ± 13 years, 59% females, 42% HISP, 23% NHW, 35% NHB), with obesity (BMI 36.7 ± 6.2), 72% perceived themselves as having overweight and only 24% as having obesity. Among the three groups, HISP had the highest AFA score (3.6 ± 1.3 vs. 3.3 ± 1.1 vs. 2.1 ± 1.2, *p* < 0.001), NHW had the highest SSI-B (1.2 ± 1.1 vs. 2.0 ± 1.4 vs. 1.2 ± 1.4, *p* < 0.001), and NHB had the lowest WBIS (2.7 ± 1.2 vs. 3.0 ± 1.3 vs. 2.3 ± 1.2, *p* < 0.001) score.

**Conclusions:**

Among an ethnically diverse U.S. cohort of adults with obesity, most participants perceive themselves as having overweight and not obesity. HISP exhibited greater aversion and prejudices towards people with obesity, NHW reported more stigmatizing situations and NHB the lowest internalization of negative bias. These findings should be incorporated when developing or implementing culturally appropriate educational or interventional programs for people with obesity.

## Introduction

Weight stigma represents a pervasive and increasingly recognized form of prejudice that significantly impacts individuals with obesity. Despite growing awareness of obesity as a complex, multifactorial chronic disease [1, 2], societal attitudes continue to perpetuate harmful stereotypes that blame and marginalize individuals based on body weight and size [3]. Notably, as obesity rates rise, weight prejudice also increases [4].

Weight bias, weight stigma, experienced weight stigma, and internalized weight bias represent interconnected yet distinct constructs that collectively describe the psychological and social mechanisms of weight-based prejudice [4]. Weight bias encompasses negative attitudes and beliefs, while weight stigma extends to active discrimination and social devaluation [4]. Experienced weight stigma refers to direct, observable instances of prejudice, such as workplace marginalization, healthcare disparities, and social exclusion [4]. Internalized weight bias represents the deep personal process by which individuals incorporate and accept negative societal narratives about their bodies, leading to profound psychological consequences [4]. Individuals experiencing weight-based biases face increased risk of depression, anxiety, eating disorders, as well as reduced quality of life and compromised physical health outcomes [1, 5–9]. Moreover, weight stigma can create a cyclical pattern that drives weight gain [10], and exacerbates the chronic disease care, as stigmatized individuals may avoid healthcare interactions, exercise, and social engagement [7, 11].

Understanding weight stigma is complex, as cultural contexts, body ideals, and historical experiences of prejudice interact with weight-related experiences. Different ethnic groups may have varying cultural interpretations of body size, aesthetic preferences, and social norms that influence weight stigma experiences. Existing research on weight stigma across ethnic groups remains limited, with most studies predominantly focusing on White populations [11, 12]. Consequently, we conducted a survey study to compare 1) weight self-perception, 2) weight-related aversion and prejudices, 3) stigmatizing experiences, and 4) internalization of negative weight bias in a diverse ethnic cohort in the U.S.

## Methods

### Study design and data collection

In this cross-sectional observational survey study, we analyzed data from 296 participants interviewed at two academic outpatient medical clinics in Atlanta, Georgia (Grady Memorial Hospital - Emory University), and Miami, Florida (University of Miami) between December 2022 and March 2024. This study is part of an initiative by the Spanish Society for the Study of Obesity (Sociedad Española para el Estudio de la Obesidad – SEEDO) aimed at investigating bias, stigmatization, and negative attitudes related to obesity across different countries [11, 13].

### Participants

Adults ≥ 18 years of age with a body mass index (BMI) ≥ 30 kg/m^2^, able to provide informed consent and had available laboratory data completed within the prior 6 months. We excluded those with a life expectancy of <6 months, pregnancy, and cognitive impairment that would prevent the individual from comprehending the study. The Institutional Review Boards of the University of Miami and Emory University approved the study protocol. We invited patients presenting for their routine medical visits at the academic endocrine practice of the University of Miami and at the Diabetes Center, in Grady Memorial Hospital. Among 422 participants contacted, 122 patients refused to participate in the study. A total of 4 participants were excluded due to not having available laboratory data from the prior 6 months (response rate of 71.1%, completion rate of 98.7%). Consequently, the final sample consisted of 296 participants.

### Objectives

Our primary objective was to compare 1) Weight self-perception, 2) weight-related aversion and prejudices with Anti-Fat Attitudes (AFA), 3) stigmatizing experiences with Brief Stigmatizing Situations Inventory (SSI-B), and 4) internalization of negative weight bias with Weight Bias Internalization Scale (WBIS) scores between groups. Secondary objectives included examining the relationships between clinical and demographic characteristics and the survey scores.

### Procedures

Trained clinical staff measured participants’ height, weight, and waist circumference using standardized protocols before conducting the surveys. BMI was calculated as weight (kg) / [height (m)]^2^. Self-reported demographic characteristics were collected as part of the surveys (age, gender, race, ethnicity, country of origin, time of residence in the U.S., education, marital status, income, employment, and health insurance). Electronic medical records were queried to obtain laboratory data.

### Surveys

Participants completed three validated instruments on the same day:

Anti-Fat Attitudes (AFA) Scale: A 13-item scale grouped into three subscales (dislike, fear of fat, willpower) measuring antipathy toward people with obesity, fear of obesity, and perceived willingness to control weight [14]. Responses ranged from 0 (“not at all agree”) to 9 (“strongly agree”).

Brief Stigmatizing Situations Inventory (SSI-B): A 10-item scale assessing lifetime experiences of weight-related stigma across various societal domains [15]. Items are scored on a 10-point scale (0 = “never”, 1 = “once in a lifetime”, 2 = “many times in life”, 3 = “once a year”, 4 = “quite a few times a year”, 5 = “once a month”, 6 = “quite a few times a month”, 7 = “once a week”, 8 = “quite a few times a week”, and 9 = “daily”).

Weight Bias Internalization Scale (WBIS): An 11-item questionnaire measuring the internalization of negative weight stereotypes [16]. Responses are measured on a 7-point Likert scale, ranging from “strongly disagree” to “strongly agree.”

### Statistical analysis

The initial analysis consisted of testing variables by three distinct groups: Hispanic, Non-Hispanic Whites, and Non-Hispanic Blacks. Continuous variables are reported as mean (standard deviation) and categorical variables are reported as frequency count (frequency percentages). Continuous variables were tested using ANOVA methods, whereas categorical variables were tested using Chi-Square tests. All testing was conducted at a threshold level of alpha = 0.05. After initial analysis, multivariate regression analysis was performed on outcome variables of interest. Outcome variables of interest included the scores of three surveys: 1) Anti-Fat Attitudes Scale, 2) Brief Stigmatizing Situations Inventory, and 3) Weight Bias Internalization Scale. The PROC GLM function in SAS was utilized to estimate multivariate hierarchal regression models on outcomes relative to ethnic groups. Adjustments were then made based on age, gender, education level, income, and BMI (supplemental table). Reference variables for categorical variables are as follows: race group (Hispanic), gender (Male), and education (Graduate Degree). All data analyses were conducted in SAS Version 9.4.

## Results

### Participants characteristics

Among 296 participants completing the surveys, the mean age was 54.8 ± 13 years, 59% were females, with a mean BMI of 36.7 ± 6.2 kg/m^2^. Hispanics represented 42% (*n*: 123), non-Hispanic Whites 23% (*n*: 68), and Non-Hispanic Blacks 35% (*n*: 105) of the study group (see Table 1).

**Table 1:** Demographic and clinical characteristics among adults with obesity.

	Overall (*N* = 296)	HISP (*N* = 123)	NHW (*N* = 68)	NHB (*N* = 105)	*p*-value
**Age**, years	54.88 (12.97)	55.37 (12.97)	54.69 (13.66)	54.42 (12.61)	0.878
**Sex**, Female, *n* (%)	174 (59)	72 (58)	31(46)	71(68)	0.016*
**Education**, High School or less, *n* (%)	123 (42)	46 (37)	10 (15)	67 (64)	<0.0001*
Technical Degree	57 (19)	23 (20)	12(17)	22 (21)	
University or Graduate school	116 (39)	54 (44)	46 (68)	16(15)	
**Insurance**, Government, *n* (%)	121 (41)	51 (41)	19 (28)	51 (49)	<0.0001*
Private	142 (48)	57 (46)	47(69)	38 (36)	
Uninsurance/County	33 (11)	15 (13)	2 (3)	16 (15)	
**Weight**, kg	104.30 (23.17)	99.43 (19.23)	106.72 (24.98)	108.42 (25.27)	0.0075*
**BMI**, kg/m^2^	36.73 (6.24)	36.40 (5.12)	35.93 (7.12)	37.65 (6.8)	0.086
**Waist Circumference**, cm	118.23 (14.06)	116.72 (12.5)	115.32 (13.24)	121.90 (15.58)	0.0069*
**Diabetes**, *n* (%)	223 (74.58)	87 (70)	43 (63)	90 (86)	0.02*

Values reported are as means ± standard deviation unless otherwise specified.

*HISP* Hispanic, *NHW* non-Hispanic White, *NHB* non-Hispanic Black.

*denotes statistically significant

**Fig. 1:**
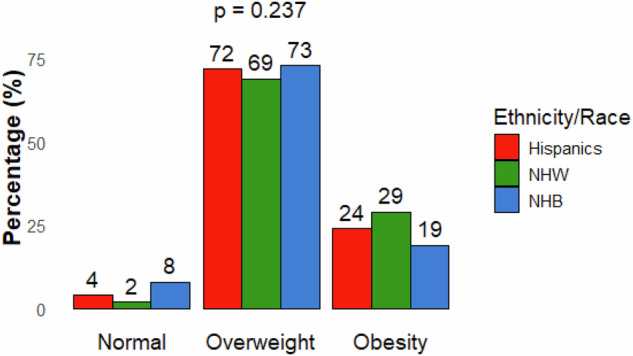
Perceived weight status among Hispanic, non-Hispanic White, and non-Hispanic Black individuals with obesity in the U.S. *All participants had a BMI ≥ 30 kg/m². Mean BMI (kg/m²): Hispanics = 36.40, NHW = 35.93, NHB = 37.65. NHW non-Hispanic White, NHB non-Hispanic Black.

Self-perception of having overweight was reported by 72% (*n*: 88) of Hispanics, 69% (*n*: 47) of Non-Hispanic Whites, and 73% (*n*:77) of Non-Hispanic Blacks. Self-perception of having obesity was reported by 24% (*n*: 30) of Hispanics, 29% (*n*: 20) of Non-Hispanic Whites, and 19% (*n*:20) of Non-Hispanic Blacks (shown in Fig. 1).

### Surveys scores

#### Anti-fat attitudes, total score (AFA, weight-related perception and prejudices)

**Fig. 2:**
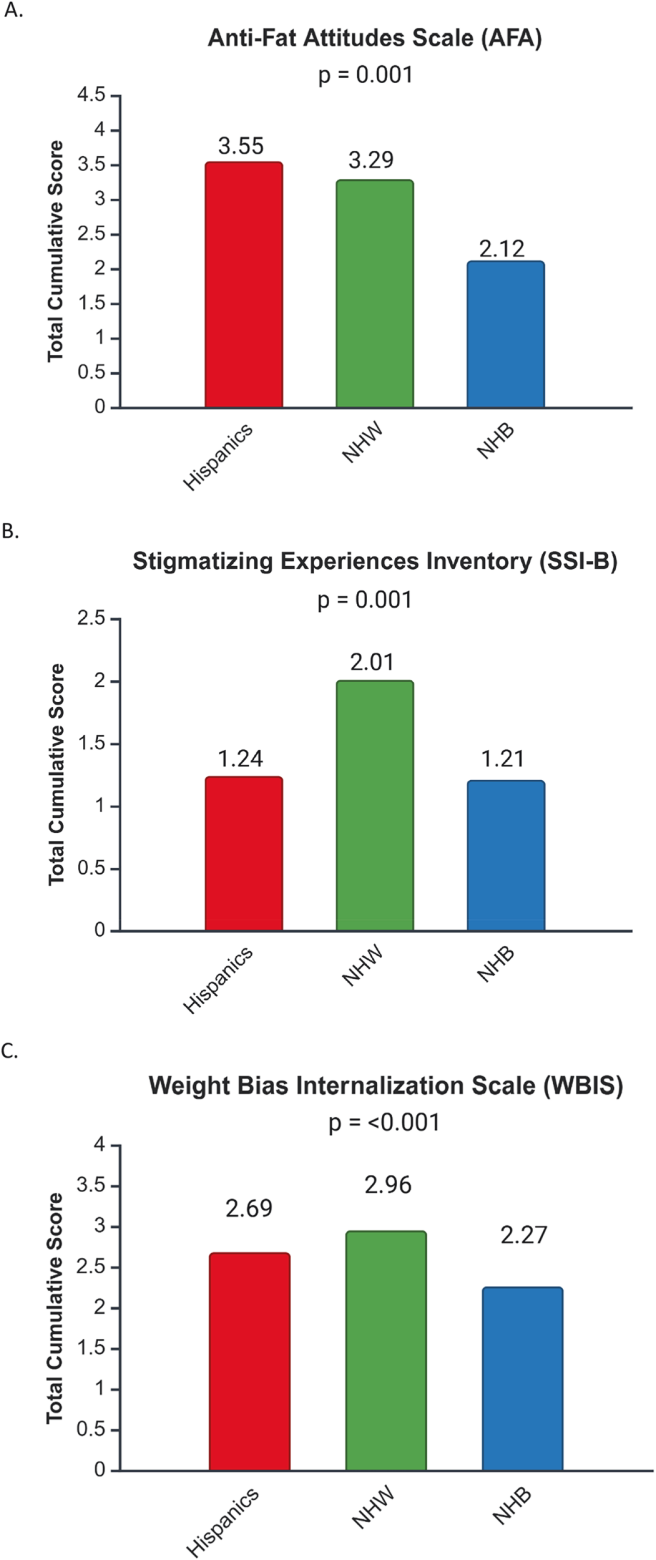
Survey results across racial/ethnic groups. NHW non-Hispanic White, NHB non-Hispanic Black. **A** Anti-fat Attitudes Scale. **B** Stigmatizing Experiences Inventory (SSI-B). **C** Weight Bias Internalization Scale (WBIS).

Hispanic individuals had higher total scores (3.55 ± 1.25) compared to non-Hispanic White (3.29 ± 1.14) and non-Hispanic Black individuals (2.12 ± 1.24, *p* < 0.0001) (shown in Table 2, Fig. 2).

**Table 2:** Anti-fat attitudes (AFA) survey: total scores and subscale items.

	HISP (*N* = 123)	NHW (*N* = 68)	NHB (*N* = 105)	*p*-value
AFA total score (mean±SD)	3.55 (1.25)	3.29 (1.14)	2.12 (1.24)	<0.0001*
AFA subscales				
Dislike component	1.96 (1.22)	1.84 (1.155)	0.877 (0.977)	<0.0001*
I really don’t like fat people much.	2.70 (2.65)	2.15 (1.89)	0.8 (1.4)	<0.0001*
I don’t have many friends that are fat.	3.78 (3.0)	2.93 (2.4)	2.02 (2.71)	<0.0001*
I tend to think that people who are overweight are a little untrustworthy.	1.20 (1.26)	1.4 (1.3)	0.61 (1.19)	<0.0001*
Although some fat people are surely smart, in general, I think they tend not to be quite as bright as normal-weight people.	1.02 (0.96)	1.5 (1.7)	0.69 (1.54)	<0.0001*
I have a hard time taking fat people too seriously.	1.30 (1.27)	1.36 (1.14)	0.62 (1.25)	<0.0001*
Fat people make me somewhat uncomfortable.	1.46 (1.61)	1.65 (1.6)	0.52 (1.05)	<0.0001*
If I were an employer looking to hire, I might avoid hiring a fat person.	2.28 (2.35)	1.94 (1.86)	0.81 (1.44)	<0.0001*
Fear of fat component	5.90 (2.51)	5.0 (2.3)	3.23 (2.83)	<0.0001*
I feel disgusted with myself when I gain weight.	5.49 (3.05)	5.38 (2.78)	2.93 (3.35)	<0.0001*
One of the worst things that could happen to me would be if I gained 25 pounds	5.31 (3.2)	3.66 (2.6)	3.04 (3.29)	<0.0001*
I worry about becoming fat.	6.9 (2.87)	5.96 (2.91)	3.72 (3.42)	<0.0001*
Willpower issues component	4.88 (2.21)	4.94 (2.18)	3.92 (2.5)	0.0025*
People who weigh too much could lose at least some part of their weight through a little exercise.	6.05 (2.84)	6.12 (2.67)	5.36 (3.29)	0.3360
Some people are fat because they have no willpower.	5.04 (2.92)	5.15 (3.13)	3.64 (3.17)	0.0003*
Fat people tend to be fat pretty much through their own fault.	3.56 (2.82)	3.54 (2.53)	2.76 (2.78)	0.0084*

Values reported are as means ± standard deviation unless otherwise specified.

*AFA* Antifat Attitudes Scale, *HISP* Hispanic, *NHW* non-Hispanic White, *NHB* non-Hispanic Black.

*denotes statistically significant

#### AFA subscales

1a) AFA Dislike component: Hispanic individuals scored highest (1.96 ± 1.2), followed by non-Hispanic White (1.84 ± 1.16) and non-Hispanic Black individuals (0.88 ± 0.98, *p* < 0.0001).

1b) AFA Fear of Fat component: Hispanic individuals had the highest scores (5.90 ± 2.51), followed by non-Hispanic White (5.0 ± 2.3) and non-Hispanic Black individuals (3.23 ± 2.83, *p* < 0.0001).

1c) AFA Willpower Issues component: Non-Hispanic White individuals scored highest (4.94 ± 2.18), followed by Hispanic (4.88 ± 2.21) and non-Hispanic Black individuals (3.92 ± 2.5, *p* = 0.0025) (Table 2).

After adjusting for multiple covariates (age, sex, income, education, and BMI), non-Hispanic Black individuals had significantly lower AFA total scores compared to Hispanic individuals (β = -1.385, SE = 0.18, *p* < 0.001). There was no significant difference between non-Hispanic White and Hispanic individuals (β = -0.210, SE = 0.21, *p* = 0.334). Additionally, a statistically significant negative association existed between BMI and AFA scores (β = -0.034, SE = 0.011, *p* = 0.009) (shown in Supplemental Table 1).

#### Brief Stigmatizing Situations Inventory (SSI-B, stigmatizing experiences)

Non-Hispanic White individuals had higher total SSI-B scores (2.01 ± 1.36) compared to Hispanic (1.24 ± 1.14) and non-Hispanic Black individuals (1.21 ± 1.37, *p* < 0.0001) (shown in Fig. 2).

A higher proportion of Non-Hispanic White individuals reported experiencing ‘’children loudly making comments (fun) about your weight to others” than Hispanic and non-Hispanic Black individuals (77.94% for non-Hispanic White vs. 31.14% for Hispanic and 35.23% for non-Hispanic Black adults, *p* < 0.001). Similarly, a higher proportion of non-Hispanic White individuals reported ‘’being singled out as a child by a teacher, school nurse, etc., because of your weight” (77.94% vs 31.14% for Hispanic and 25.71% for non-Hispanic Black adults, *p* = <0.0001) and ‘’having a romantic partner exploit you because she or he assumed you were desperate and would put with it” (72.05% vs. 23.77% and 23.8%, respectively, *p* = <0.0001, Table 3). Of note, non-Hispanic White individuals reported a higher number of experiences of ‘’having a doctor recommend a diet, even if did not come in to discuss weight loss” (94.11% vs. 83.78% for Hispanic and 80.95% for non-Hispanic Black adults, *p* = 0.01), and ‘’not being hired because of your weight, shape or size” (72.07% vs. 27.04% for Hispanic and 23.80% for non-Hispanic Black adults, *p* = <0.0001) (Table 3).

**Table 3:** Brief Stigmatizing Situations Inventory (SSI-B): total scores and proportion of participants reporting stigmatizing experiences.

	HISP (*N* = 123)	NHW (*N* = 68)	NHB (*N* = 105)	*p*-value
SSI-B total scores, mean (SD)	1.24 (1.14)	2.01 (1.36)	1.21 (1.37)	<0.0001*
Stigmatizing experiences, *N* (%)				
Children loudly making comments (fun) about your weight to others.	38 (31.14)	53 (77.94)	37 (35.23)	<0.0001*
Being singled out as a child by a teacher, school nurse, etc., because of your weight.	38 (31.14)	53 (77.94)	27 (25.71)	<0.0001*
Having a romantic partner exploit you because she or he assumed you were “desperate” and would put up with it.	29 (23.77)	49 (72.05)	25 (23.80)	<0.0001*
Having people assume you overeat, or binge eat because you are overweight.	74 (60.65)	57 (83.83)	52 (49.52)	0.0029*
Overhearing other people making rude remarks about you in public.	50 (40.98)	50 (73.52)	39 (37.1)	<0.0001*
Being stared at in public.	38 (31.14)	59 (86.76)	42 (40%)	<0.0001*
Having a doctor recommend a diet, even if did not come in to discuss weight loss.	101 (82.78)	64 (94.11)	85 (80.95)	0.0129*
Having family members feel embarrassed by you or ashamed of you.	44 (36.06)	50 (73.52)	23 (21.90)	<0.0001*
Not being hired because of your weight, shape or size	33 (27.04)	49 (72.07)	25 (23.80)	<0.0001*
Being glared at or harassed for taking up “too much” room.	27 (22.13)	50 (73.52)	29 (27.61)	<0.0001*

*HISP* Hispanic, *NHW* non-Hispanic White, *NHB* non-Hispanic Black, *SSI-B* Brief Stigmatizing Situations Inventory.

*denotes statistically significant

After adjusting for multiple covariates, non-Hispanic White individuals had significantly higher SSI-B scores than Hispanic individuals (β = 0.894, SE = 0.22, *p* < 0.001). There was no significant difference between non-Hispanic Black and Hispanic individuals (β = -0.136, SE = 0.19, *p* = 0.476). The SSI-B scores decreased with age (β = −0.19, SE = 0.006, *p* = 0.024). Individuals with less than high school education had significantly higher scores compared to those with a graduate degree (β = 1.134, SE = 0.32, *p* < 0.001) (shown in Supplemental Table 1).

#### Weight Bias Internalization Scale (WBIS, internalization of negative weight bias)

Hispanic (2.69 ± 1.22) and non-Hispanic White individuals (2.96 ± 1.25) had higher WBIS scores, compared to non-Hispanic Black individuals (2.27 ± 1.22, *p* = 0.0006) (shown in Fig. 2, Table 4).

**Table 4:** Weight Bias Internalization Scale (WBIS): total scores and participant scores across each item of the WBIS among groups.

	HISP (*N* = 123)	NHW (*N* = 68)	NHB (*N* = 105)	*p*-value
WBIS total scores (mean±SD)	2.69 (1.22)	2.96 (1.25)	2.27 (1.22)	0.0006*
Scale item				
As an overweight person, I feel that I am just as competent as anyone.*	1.73 (1.44)	1.38 (1.03)	1.64 (1.22)	0.1699
I am less attractive than most other people because of my weight.	3.14 (2.33)	3.44 (2.13)	2.47 (2.02)	0.0041*
I feel anxious about being overweight because of what people might think of me.	2.73 (2.22)	3.54 (2.39)	1.95 (1.75)	<0.0001*
I wish I could drastically change my weight.	5.28 (2.1)	5.48 (2.07)	4.55 (2.36)	0.0266*
Whenever I think a lot about being overweight, I feel depressed.	3.1 (2.32)	3.25 (2.09)	2.62 (1.87)	0.2079
I hate myself for being overweight.	1.84 (1.68)	2.04 (1.76)	1.68 (1.52)	0.1316
My weight is a major way that I judge my value as a person.	1.8 (1.59)	2.33 (1.87)	1.81 (1.72)	0.0055*
I don’t feel that I deserve to have a really fulfilling social life, as long as I’m overweight.	1.38 (0.99)	1.63 (1.43)	1.28 (0.99)	0.0519*
I am OK being the weight that I am.^a^	3.59 (1.61)	4.13 (1.67)	3.19 (1.69)	0.0144*
Because I’m overweight, I don’t feel like my true self.	2.87 (2.24)	3.08 (2.18)	2.2 (1.98)	0.0025*
Because of my weight, I don’t understand how anyone attractive would want to date me.	2.53 (2.05)	2.47 (2.04)	1.73 (1.6)	0.0010*

Values reported are as means ± standard deviation unless otherwise specified.

*HISP* Hispanic, *NHW* non-Hispanic White, *NHB* non-Hispanic Black, *WBIS* Weight Bias Internalization Scale.

^a^Reverse-scored.

*denotes statistically significant

These results persisted after adjusting for covariates; non-Hispanic Black individuals had significantly lower WBIS scores than Hispanic individuals (β = −0.626, SE = 0.17, *p* < 0.001). There was no significant difference between non-Hispanic White and Hispanic individuals (β = 0.386, SE = 0.20, p = 0.055). WBIS scores decreased with age (β = −0.018, SE = 0.005, *p* = 0.001), and increased with an educational level less than high school (β = 0.596, SE = 0.29, *p* = 0.043. No significant associations were found between WBIS scores and other education levels or BMI in the adjusted model (shown Supplemental Table 1).

## Discussion

The USA-OBESTIGMA study interviewed adults with obesity in two academic medical centers in Atlanta (Georgia) and Miami (Florida). We observed several important differences in weight stigma among racial and ethnic groups with obesity in the U.S. First, we noticed that despite all participants having obesity (mean BMI ~ 37 kg/m^2^), only 24% of Hispanic, 29% of non-Hispanic White, and 19% of non-Hispanic Black participants had the self-perception of having obesity. Most considered themselves having overweight, with 72% of Hispanic, 69% non-Hispanic White, and 73% non-Hispanic Black individuals. Second, Hispanic adults reported higher aversion and prejudice towards people with obesity, compared to their non-Hispanic White and non-Hispanic Black counterparts, as measured by the AFA survey. Third, non-Hispanic White individuals reported higher scores on stigmatizing experiences than Hispanic and non-Hispanic Black participants. Fourth, non-Hispanic Black individuals reported lower internalization of negative weight bias scores compared to Hispanic and non-Hispanic White individuals.

It was not surprising that there are high rates of weight misperception among minorities with a BMI ≥ 30 kg/m^2^ in the U.S. Prior studies have shown that racial and ethnic minority groups, including Hispanic and Black adults, may have a higher prevalence of misperceived weight status [13, 17, 18]. This misperception could be influenced by cultural norms surrounding body image, where a larger body size may be more socially accepted or preferred within certain communities. Additionally, differences in exposure to health education, healthcare access, and societal weight norms may contribute to these variations in self-perception [13, 17].

Racial and ethnic differences in weight-related stigma can be attributed to a variety of factors, including socioeconomic status, cultural norms, and personal experiences. Cultural attitudes towards body weight and shape can vary significantly across different racial and ethnic groups. It is well established that body size and ideal body weight are stigmatizing factors in Hispanic communities, albeit with some differences across countries of origin. For example, Caribbean communities may consider a larger and curvy body as desirable, while other groups may prefer slender body sizes or contours [18, 19]. This again highlights the associated stigma related to obesity, seen by many still as a body image disorder and not an actual chronic metabolic disease with several associated complications. Compared to Hispanic adults, non-Hispanic Black individuals had a lower AFA score, suggesting they are more accepting of people with larger body sizes. This may also explain why non-Hispanic Black individuals had the lowest scores for internalization of negative bias [20]. Recent studies also found that non-Hispanic Black individuals had lower weight bias internalization than other racial and ethnic individuals [21, 22]. Our study also found SSI-B score were lower for non-Hispanic Black individuals, and higher in non-Hispanic White females, aligning with recent studies of diverse U.S. samples [23, 24]. These findings suggest that societal and cultural perceptions of body weight play a role in weight-related stigma and bias internalization across racial and ethnic groups. These findings highlight the need for better education among patients, emphasizing the implications of obesity as a chronic disease of excess adiposity and explaining the different risk categories [25].

Our study also examined how the degree of obesity and personal characteristics influence negative feelings and stigma. The statistically significant negative association between BMI and AFA scores suggests that individuals with higher BMI might be less likely to have prejudice against other people with obesity, possibly due to a coping mechanism or resilience developed over time. Similarly, in multivariate analysis of AFA scores, we found no differences after adjusting for age and gender, but a negative correlation with type 2 diabetes.

The association of higher SSI-B scores with lower education levels highlights the compounded stigma faced by less-educated individuals [11]. One might assume that people living with a disease would be more understanding toward others having the same condition. However, this does not appear to be the case regarding anti-obesity attitudes. Higher degrees of obesity are associated with higher internalized stigma scores, which creates a challenging cycle where negative self-perception can impede effective weight management and potentially contribute to further weight gain [10]. This internalized stigma is often compounded by experiences with healthcare providers, where weight bias can lead to poor communication and inadequate care, ultimately affecting health outcomes and patients’ willingness to seek treatment [26, 27]. Guidelines emphasizes that healthcare providers must confront the widespread issue of fat bias in clinical settings, advocating for increased awareness of weight-biased attitudes and deeper understanding of weight management’s complexities among medical professionals [28].

Notably, we found that SSI-B scores decrease with age, suggesting that younger individuals are exposed to the most weight-related stigmatization experiences. This finding aligns with recent research from a cross-sectional survey of adults with obesity, which demonstrated that individuals with severe obesity before the age of 18 years were nearly three times more likely to report severe experiences of weight stigma compared to those who developed severe obesity later in life ( > 18 years of age) [24]. Similarly to their findings, we found that “*Having a doctor recommend a diet, even if did not come in to discuss weight loss”’* was the most common stigmatizing experience among the three groups [24]. Other study among university students in nursing and social work careers in the U.S. and Turkey reported similar patterns, in which younger students had worse scores [29, 30]. Furthermore, the OBESTIGMA study in Spain, recruiting over 1200 participants, with a mean age of 33.3 years, reported that younger participants had more frequent stigmatizing experiences [11]. This highlights the heightened vulnerability of younger individuals to weight stigma and shows that our society, whether in Europe or the U.S., still lacks an inclusive environment for people with obesity. These findings support the incorporation of obesity and stigma in the curriculum, not only for medical or health-related students, but also in the general population, and at early ages. We advocate for professional societies, patient advocacy groups, and society to incorporate these findings into future educational programs. Obesity stigma can lead to poor communication and inadequate care for individuals with obesity, ultimately affecting their health outcomes and willingness to seek treatment [26, 27].

The strengths of this study include a diverse patient population from two large metropolitan areas, inclusion of a diverse ethnic population, the use of multiple surveys, and the exploration of the relationship between obesity and sociodemographic variables. However, the study has a few limitations, including a smaller sample size compared to similar studies in other countries [11]. The small sample size may limit generalizability to the U.S. population. We used BMI to define obesity, which has inherent limitations. Several experts and professional societies have advocated using metrics to diagnose, stage, and manage obesity beyond BMI, including validating metrics such as waist-to-height ratio, body composition studies, and clinical/complications [18]. Although our study actively recruited participants from three specific racial/ethnic groups with balanced representation, we recognize that our Florida-based sampling, where Hispanic/Latino populations constitute a larger demographic proportion than in many other U.S. regions, may not accurately reflect the racial/ethnic distribution of the general U.S. population. This geographical concentration potentially limits the generalizability of our findings to the broader national context despite our efforts to minimize selection bias. In the future, it would be interesting to repeat our approach while incorporating other variables that more reliably address excess adiposity associated with obesity. Additionally, due to the observational nature of the study, associations cannot be interpreted as causal relationships. Lastly, the reliance on self-reported data introduces the potential for recall or social desirability bias.

## Conclusion

Our study highlights significant differences in weight bias and stigmatization among racial and ethnic groups with obesity in the U.S. Only a minority of participants accurately identified themselves as having obesity. Hispanic individuals reported higher aversion and prejudice toward individuals with obesity, non-Hispanic White individuals experienced more stigmatizing encounters, and non-Hispanic Black individuals had lower internalization of negative weight bias. Based on these findings, we urge professional societies, patient advocacy groups, and public health policymakers to develop educational programs that actively address and reduce weight bias and stigma.

## Supplementary information

Supplemental Table 1

## Data Availability

Some or all datasets generated during and/or analyzed during the current study are not publicly available. A deidentified file with data will be available to researchers upon reasonable request, after submitting an IRB approved protocol, and after 24 months of publication.
