# Supplementary material for: Weight stigma among diverse ethnic groups with obesity in the U.S.: the USA-OBESTIGMA study
Source: Int J Obes (Lond). 2026 Feb 14;50(5):1027–34. doi: 10.1038/s41366-026-02028-z (PMC13226056; doi:10.1038/s41366-026-02028-z)
Supplement: Supplementary file 1 — Supplemental Table 1 [file 41366_2026_2028_MOESM1_ESM.docx]

| Variable | AFA |  | SSI-B |  | WBIS |  |
| --- | --- | --- | --- | --- | --- | --- |
|  | *β* (SE) | *P-value* | *β* (SE) | *P-value* | *β* (SE) | *P-value* |
| Ethnicity/race group |  |  |  |  |  |  |
| Hispanic (reference) | - | - | - | - | - | - |
| Non-Hispanic White | -0.210 (0.21) | 0.334 | 0.894  (0.22) | <0.001* | 0.386  (0.20) | 0.055* |
| Non-Hispanic Black | -1.385 (0.18) | <0.001* | -0.136 (0.19) | 0.476 | -0.626 (0.17) | <0.001* |
| Age | 0.005 (0.006) | 0.444 | -0.19 (0.006) | 0.024* | -0.018 (0.005) | 0.001* |
| Gender |  |  |  |  |  |  |
| Male (reference) | - | - | - | - | - | - |
| Female | -0.021  (0.16) | 0.898 | 0.106  (0.16) | 0.526 | 0.233  (0.15) | 0.12 |
| Income | -6.73e-7 (1.14e-6) | 0.553 | -1.26e-6 (1.15e-6) | 0.274 | -9.00e-7 (1.04e-6) | 0.389 |
| Education |  |  |  |  |  |  |
| No Education | -0.562  (0.90) | 0.535 | 1.093  (0.91) | 0.234 | -0.301 (0.83) | 0.71 |
| Less than high school | 0.001  (0.31) | 0.996 | 1.134  (0.32) | <0.001* | 0.596  (0.29) | 0.043* |
| High School Diploma/GED | -0.113  (0.25) | 0.651 | 0.032  (0.25) | 0.899 | 0.168  (0.23) | 0.465 |
| Technical/associate’s degree | 0.123  (0.25) | 0.629 | 0.344  (0.25) | 0.185 | 0.202  (0.23) | 0.390 |
| University Degree (reference) | -0.180  (0.26) | 0.491 | 0.316  (0.26) | 0.233 | -0.074 (0.24) | 0.755 |
| BMI | -0.034  (0.01) | 0.009* | 0.016 (0.013) | 0.218 | -0.007 (0.012) | 0.535 |

**Supplemental Table 1:** Multivariate Analysis of AFA, SSI-B, and WBIS Scores, adjusted by age, sex, income, education, and BMI.

*AFA = Anti-fat Attitudes Scale, SSI-B = Brief Stigmatizing Situations Inventory, WBIS = Weight Bias Internalization Scale. β (SE) represents the standardized regression coefficient and its standard error. Reference categories are indicated.*
